# All Effects of Psychophysical Variables on Color Attributes: A Classification System

**DOI:** 10.1371/journal.pone.0119024

**Published:** 2015-04-10

**Authors:** Ralph W. Pridmore, Manuel Melgosa

**Affiliations:** 1 Central Houses P/Ltd, 8c Rothwell Rd, Turramurra, Sydney, NSW, 2074, Australia; 2 Department of Optics, University of Granada, Granada, E-18071, Spain; University of Western Australia, AUSTRALIA

## Abstract

This paper reports the research and structuring of a classification system for the effects of psychophysical variables on the color attributes. A basic role of color science is to psychophysically specify color appearance. An early stage is to specify the effects of the psychophysical variables (as singles, pairs, etc) on the color attributes (as singles, pairs, etc), for example to model color appearance. Current data on effects are often scarce or conflicting. Few effects are well understood, and the practice of naming effects after their discoverer(s) is inadequate and can be confusing. The number and types of possible effects have never been systematically analyzed and categorized. We propose a simple and rigorous system of classification including nomenclature. The total range of effects is computed from the possible combinations of three psychophysical variables (luminance, dominant wavelength, purity) and six color attributes (lightness, brightness, hue, chroma, colorfulness, saturation) in all modes of appearance. Omitting those effects that are normally impossible to perceive at any one time (such as four- or five-dimensional colors), the total number perceivable is 161 types of effects for all modes of appearance. The type of effect is named after the psychophysical stimulus (or stimuli) and the relevant color attribute(s), e.g., Luminance-on-hue effect (traditionally known as Bezold-Brucke effect). Each type of effect may include slightly different effects with infinite variations depending on experimental parameters.

## Introduction

A fundamental aim of color science is to relate the perception of color to the physical or psychophysical stimuli, in order to understand color (as basic science) and apply the knowledge to commerce and industry (as applied science). The objective is to know how every physical or psychophysical variable influences perceived color, and the reverse: how color depends on each variable [1]. From such knowledge, every nuance of perceived color could in theory be predicted from a finite set of psychophysical variables. The variables are principally the three classical psychophysical variables of luminance, dominant (or complementary) wavelength, and purity [1,2], but also such experimental parameters as sample size, homogeneity, and background may be considered.

The color attributes are principally lightness/brightness, hue, and chromaticness (a broad term for chroma, colorfulness, or saturation) [1,2]. The data base necessary to formulate relations between these color attributes and the psychophysical variables would be large and require a classification system to group, relate and name the effects so that one is not confused with another and relations with each other may be determined. To research and structure such a classification system is the purpose of this paper. Classification of basic phenomena (such as psychological/perceptual effects of physical stimuli) is an essential task in any science, and normally executed in the early stages. A well known example, though of much larger scale, is the classification and binominal nomenclature given by the botanist Linnaeus [3] to biological species.

Perceived color is widely accepted to be three dimensional, or definable in a three dimensional space, as shown, for example, by the numerous color-order systems and related color atlases in use. These three dimensions (let us call them psychological to differentiate from psychophysical) are lightness (or its alternative perception, brightness), hue, and chromaticness. Such perceptions are also known as color attributes, of a total six (lightness, brightness, hue, saturation, colorfulness, and chroma). Although color is three dimensional, a single dimension may have alternative perceptions. For example, the lightness dimension may be perceived as either lightness or brightness. At any one time, the dimension may be perceived as either of those variants, but not more than one variant at one time since color is limited to three dimensions at one time.

If a critical proportion of the required data and relations were gained, the remaining details may possibly be estimated by logic and math. The critical proportion may be defined as sufficient to establish the effects of the psychophysical variables (and combinations thereof) on each of the color attributes for the average color-normal person. Such a collection of basic effects may be envisaged as a system of interrelated formulas and theoretical color spaces which enable (1) predicted color matching and color mixture, (2) a numerical system of color measurement, and (3) assessment of color difference and color appearance in its major attributes. These three (1–3 above) are the basic abilities required of color science [2,4].

The reader will have recognised that abilities (1) and (2) comprise the system known as basic colorimetry [5], and that ability (3) represents the system known as advanced colorimetry [2,4,6]. Ability (3) is as yet only partly attained. Its shortfall lies in three areas: (a) first and foremost, data on the stimulus effects on color (say, *stimulus-and-effect* data); (b) formulated relations in the stimulus-and-effect data; and (c) interrelations between those effects and relations. For example, if formulated relations exist between purity and hue [7,8,9], and between purity and lightness, what is the relationship between hue and lightness for a given level of purity, so one can calculate how a change in hue may affect lightness? In part, this sort of knowledge of interrelationships already exists in basic colorimetry, such that a change in chromaticity coordinates (which correlate with interrelationships between dominant wavelength and purity) gives easily computable shifts in dominant wavelength and purity. Clearly, some methods of basic colorimetry, e.g., chromaticity diagrams, may be applied to advanced colorimetry, e.g., as (approximately) uniform color appearance spaces, and the latter tools should directly relate to the former to provide an integrated colorimetry.

A fully integrated basic and advanced colorimetry may allow the calculation, from an increment in a psychophysical variable or experimental parameter, of the consequent increments in any or all color attributes (e.g., incremental shifts in hue and lightness). Given such stimulus-and-effect formulated relations, the system may predict (and illustrate in theoretical color spaces) effects on color attributes from not only physical or psychophysical variables but psychological variables (i.e., color attributes), such as the effect of variable lightness on chroma or hue. Progress towards fully integrated colorimetry would be assisted by systematic collection and collation of stimulus-and-effect data. Inversely, progress would be hampered by careless and undisciplined gathering, reporting, and sorting of data.

Currently there is no standard system for measuring, reporting, naming, or classifying the considerable amount of stimulus-and-effect data gathered over the past century and more. Experiments have been measured in psychophysical terms (e.g., luminance) or sometimes in psychological terms (e.g., lightness), and thus cannot be directly related. Generally, effects have been named after the discoverer (e.g., Abney), even if his original experiment and results were quite different from modern data on the effect. Naming after the discoverer may be adequate if relatively few effects exist and if only one effect is discovered by the same person. However, the present paper identifies 161 types of effects. The current data-bank on a given effect may comprise few or no other experiments than the original, or if several experiments do exist (e.g., the Abney effect), the data sets may conflict.

In summary, part of the problem in the current system is inadequate stimulus-and-effect data, agreed between sufficient data sets. Another part of the problem is inadequate classification and nomenclature, both of effects and of experimental parameters. And finally, another part is that formulated relations use inappropriate or mixed metrics (mixing psychophysical and psychological terms). These problems are further discussed below.

Color science was firmly established as a psychophysical science in early 20^th^ century, due largely to groundwork and proposals by the Optical Society of America. Their pioneering work, The Science of Color [1], noted in Chapter 7 that the effects of varying luminance, purity, and dominant wavelength on the color attributes needed to be investigated and specified, and that these relationships depended on such parameters as visual field, surround chromaticity and luminance, and cognitive factors known as modes of appearance, e.g., illuminant (or aperture) mode and object mode. Fifty years later, those effects and their dependence on the various parameters remain inadequately defined [10].

Overall, the available data (see below) on the effects of the psychophysical variables are rather scarce or, in some cases, conflicting and inadequately classified. Meanwhile, modeling of color appearance has proceeded apace despite the scarcity of data, particularly data agreed between various data sets. There seems now to be a growing recognition of the need for a better data-base including detailed experimental parameters [10,11,12]. The CIE [13,14] has recently published guides to parameters and parametric effects for color difference experiments. These are similar to those for color appearance experiments, where the major parameters are luminance level, illuminant, surround and background, sample size and shape, surface characteristics, and illumination geometry [4].

This paper proposes a simple and comprehensive classification and nomenclature system for the effects of the physical or psychophysical variables (and their combinations) on the color attributes (and their combinations), and theoretically determines the full gamut of possible effects. This is the first systematic attempt to do so. Later data and analyses may indicate our proposal requires modification. Classification (including naming) encourages standard practice and has the potential to clarify and differentiate the various types of effects, and to indicate those areas requiring further research. An early incomplete version of our system was reported in [15] and was briefly outlined in [16].

No aspect of color appearance can be considered in isolation. Variance of any psychophysical variable simultaneously affects the perception of all color attributes, since in many cases one attribute (e.g., lightness) cannot vary without changing the others (e.g., hue and chroma). This is well demonstrated in a plot of Munsell data in the CIE 1931 chromaticity diagram [2,5], where for example a change of Value (or lightness) causes changes in loci of constant hue and constant chroma. This general interrelationship is problematic to understanding (and subsequently modeling) color appearance until sufficient data on the effects are gained and formulated.

### Well-Known Effects

Without attempting a review [16], the best-known effects are briefly described below by their traditional names together with references to primary experimental data (but not to articles such as algorithms predicting the data). Detailed descriptions and supporting graphs are given by Fairchild [4].

The Bezold-Brucke effect comprises the perceived hue shift of a sample with varying intensity or luminance. It is supported by more experimental data sets than perhaps any other effect. It is the earliest recorded effect [17], later detailed by Purdy [18] and other experimenters [19–28].

The Abney effect comprises the hue shift of a sample with varying purity. The original data [7] are amplified and generally supported by later experimenters [8, 9, 26,29–30]. (Ref. [26] reports both Abney and Bezold-Brucke effects.)

The Brightness:Luminance ratio (or Helmholtz-Kohlrausch effect) is a complex effect, mainly comprising the change in perceived brightness of colors/lights of varying dominant wavelength but of the same luminance [31–34]. The lowest B:L ratio is for yellow about 575 nm, and the highest ratio is at the spectrum ends (blue and red hues), indicating that B:L ratio is approximately proportional to saturation.

The Hunt effect comprises the commonly observed increase in colorfulness of a sample with increasing luminance [35].

The Stevens effect comprises the increase in brightness (or lightness) contrast of a sample with increasing luminance [36].

The Bartleson-Breneman Equations (related to the Stevens effect) is a term referring to their experimental data which showed that image contrast increases from raising surround luminance from dark to light [37].

The Helson-Judd effect comprises achromatic samples, on a white background of Munsell Value 5, exhibiting chromaticity when observed under near monochromatic illumination. Samples lighter or darker than the background show the same hue or the complementary hue, respectively, as the illumination [38,39]. The Helson-Judd effect is rarely if ever seen outside the laboratory [4], but the rather similar “colored shadows” effect [16] is commonly observed: shadows thrown by a near-monochromatic light source are seen as the complementary hue (e.g., magenta) to the light source (e.g., green).

Simultaneous contrast (or chromatic induction) is the phenomenon whereby a sample shifts its color appearance when its background color is changed [40–42]. The shift is generally in the direction complementary to the background. For example, a lighter background makes a given grey sample appear darker, or a blue background makes a grey sample appear yellowish (the complementary hue).

Crispening is a complex form of simultaneous contrast. It is the increase in perceived magnitude of color differences between two samples (in lightness or chromaticity) when the background to the samples is similar in lightness or chromaticity to the samples [43–45].

Spreading is the apparent mixing of a sample’s color with its background color, and occurs for smaller samples or thin stripes, i.e., high spatial frequency [46,47]. As the samples become thinner, their color fuses with the background color. A related effect is neon spreading [47], which includes a transparency perception.

## Preliminary Considerations

The measurement and reporting of effects may take various forms. Several options have been utilised in previous studies. For example, researchers may report results/effects of the physical and psychophysical variables upon the color attributes, say hue shift, in (a) psychological or psychometric terms such as CIELAB hue-angle, or (b) psychophysical terms, such as dominant wavelength (given in nm), or (c) psychophysical/colorimetric terms, such as chromaticity coordinates. The use of psychophysical terms (e.g., dominant wavelength, or chromaticity coordinates) appears preferable for reasons of accurate measurement of the perceived attribute increment, rather than measured in psychological terms which depend on the selected color appearance system or space. But eventually the perceived attribute increment must be converted to some psychologically meaningful term, such as hue-angle or hue quadrature [48] in an approximately uniform color space, since dominant wavelength, for instance, does not change uniformly with perceived hue shift.

Clearly, an understanding of color appearance requires data on the effects of the physical variables on color, but the former do not have a directly measurable or consistent effect on color perception. Hence in early 20^th^ century color science was agreed internationally to be a psychophysical science (psychophysics being intermediate to physics and perception; see, Appendix 1 - Definitions). The three variables (luminance, dominant wavelength, and purity) were proposed by the Colorimetry Committee of the Optical Society of America (Chapter 7 of [1]) in 1922 and accepted by international bodies (including the International Commission on Illumination, CIE, and the International Engineering Society) as the psychophysical bases of the chromaticity diagram in the CIE 1931 colorimetric system (see also Fig. five, page 67 of [1] which relates these psychophysical bases with the psychological color attributes). This chromaticity diagram was based on color matching functions derived from extensive psychophysical experiments. A given color stimulus in the CIE chromaticity diagram can be specified formally in two ways: by the chromaticity coordinates x, y, and the given relative luminance Y; or by the psychophysical variables dominant wavelength, purity, and the given relative luminance Y (or luminance factor). Hence, these psychophysical variables are commonly taken as direct psychophysical stimuli to the color attributes, for example in experiments on the effects of the former on the latter.

Thus the true physical variables (i.e. the spectral radiance or the spectral power distribution of a stimulus) were converted by psychophysical formulas (e.g. the definitions of CIE tristimulus values) to three psychophysical variables (including the photometric variable, luminance), but the precise effects of the psychophysical variables on color attributes is still a work in progress (to which the present paper is dedicated). The term psychophysical may be taken to broadly encompass photometric or colorimetric terms, all of which derive from psychophysical experiments and formulas.

**Fig 1:**
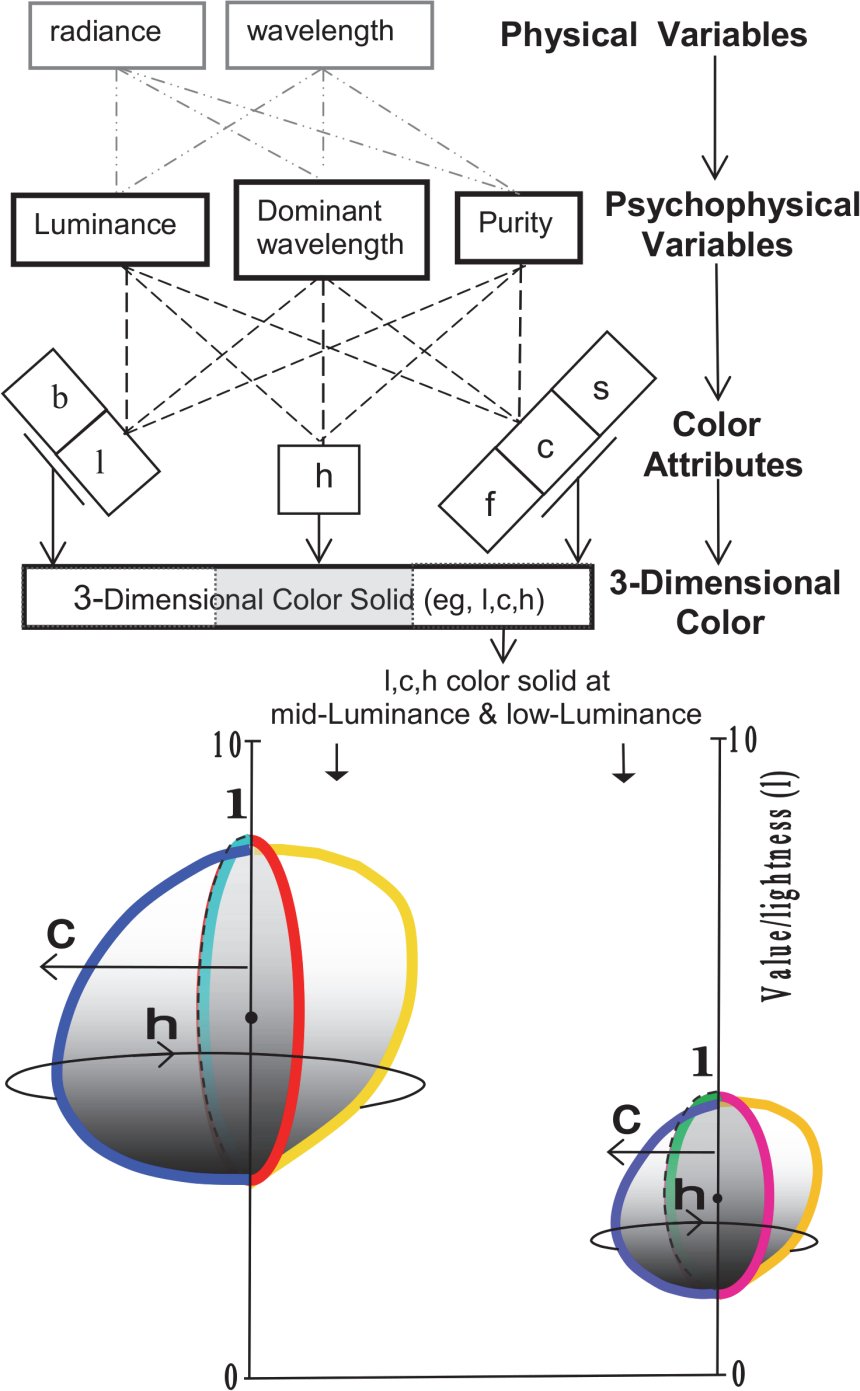
Schema of progress from physical variables through psychophysical variables and color attributes to 3-dimensional color and its maximum 6 color attributes. The attributes are lightness, brightness, hue, saturation, chroma, colorfulness, labeled l, b, h, s, c, f. Three groups of similar attributes each relate to one of 3 color dimensions, i.e., lightness/ brightness, hue, or chromaticness. One attribute represents one dimension at a time. The 3-D object color shown here has attributes l,h,c. The 9 possible effects of 3 psychophysical variables on 3 color attributes are shown as 9 dashed lines (see Table 1). If saturation applied, dashed lines would meet on “s” rather than “c”. The 3-D color solid (showing 4 of many hue planes) shows effects of raising Luminance Y (from 0.06Y to 0.3Y), roughly doubling both lightness/Value (3 to 6) and chroma (Figs III-VI(6.6.1) of [2],and Fig. 5.1 of [4]).

Fig. 1 schema shows the two physical variables (radiance and wavelength), the three classical psychophysical variables (luminance, dominant wavelength, purity) denoted by initials L, D, P, and the six color attributes (brightness, lightness, hue, and the three forms of chromaticness—saturation, chroma, colorfulness) presently recognized in color appearance science. (See Appendix 1 - Definitions) Color-order systems such as Munsell, NCS, and DIN generally define a color by a minimum of three color attributes, but a maximum of six attributes can be perceived or used to define the color appearance of some but not all colors, depending on the selected mode of color appearance. The mode of appearance assumed in Fig. 1 is object color, the most frequently encountered in natural scenes. The hypothetical color illustrated in Fig. 1 is defined by the requisite minimum three attributes, which will be designated here as lightness, hue, and chroma (as in the widely-used Munsell color-order system), and denoted by lower case letters l, h, c. The 3x3 matrix of the 3 psychophysical variables and these 3 color attributes gives nine combinations or “effects,” shown as dashed lines in Fig. 1.

**Table 1:** Class I effects (total 18), each consisting of one psychophysical variable (of a total 3) on one color attribute (of a total 6), listed by the effect name and its abbreviation in parentheses, for all modes of appearance.

Class I	Luminance (L)	Dominant Wavelength (D)	Purity (P)
lightness (l)	Luminance-on-lightness (Ll)	Dom wavelength-on-lightness (Dl)	Purity-on-lightness (Pl)
brightness (b)	Luminance-on-brightness (Lb)	Dom wavelength-on-brightness (Db)	Purity-on-brightness (Pb)
hue (h)	Luminance-on-hue (Lh)	Dom wavelength-on-hue (Dh)	Purity-on-hue (Ph)
chroma (c)	Luminance-on-chroma (Lc)	Dom wavelength-on-chroma (Dc)	Purity-on-chroma Pc)
colorfulness (f)	Luminance-on-colorfulness (Lf)	Dom wavelength-on-colorfulness (Df)	Purity-on-colorfulness (Pf)
saturation (s)	Luminance-on-saturation Ls)	Dom wavelength-on-saturation (Ds)	Purity-on-saturation (Ps)

Abbreviations generally represent initial letters of three psychophysical variables (L, D, P, in upper case) and six color attributes (l for lightness, b for brightness, h for hue, c for chroma, f for colorfulness, s for saturation, in lower case). Note P denotes colorimetric purity unless otherwise specified. Some common or traditional names of effects are: Stevens effect for Ll or Lb; Helmholtz-Kohlrausch effect (or Brightness:Luminance ratio) for Dl or Db, and also for Pl and Pb; Hunt effect (of Luminance-on-colorfulness) for Lf; Bezold-Brucke effect for Lh; and Abney effect for Ph.

These nine very common effects (dashed lines in Fig. 1) may be represented by Eq. (1) where ∆ denotes a quantified incremental difference, and → denotes “stimulates”. E.g., "∆ L→ ∆ h” means “a quantified increment in L stimulates a quantified increment in h”.

ΔL→Δl,Δh,Δc (1A)

ΔD→Δl,Δh,Δc (1B)

ΔP→Δl,Δh,Δc (1C)

Another option sometimes used is to report the effect of a color attribute (e.g., lightness) on another color attribute (e.g., hue), as represented by Eq. (2).

∆ l → ∆ h, ∆ c (2A)

∆ h → ∆ l, ∆ c (2B)

∆ c → ∆ l, ∆ h (2C)

However, Eq. (2) data and relations may be derived indirectly from data in Eq. (1), but not the reverse. Hence, effects measured in terms of Eq. (1) are generally preferable to those of Eq. (2). Eq. (1) relationships should, we feel, be researched and established before, and as a basis for, those of Eq. (2), though data on the latter are certainly required to determine the attributes’ interrelationships.

To avoid complications and allow basic science deductions, it is desirable that when experiments measure the effect of a psychophysical variable (e.g., purity) on a color attribute (e.g., hue), the other psychophysical variables be held constant [4]. In this way, clear deductions may be made of single variables’ effects on color attributes. The purpose is to specify, and thereby formulate and predict, the effect of physical or psychophysical stimuli on color appearance. Sometimes however, effects are reported in mixed terms, e.g., the effect of purity on hue may be measured with perceived lightness (rather than luminance) held constant. From such results, one cannot directly deduce the effect of the psychophysical variable on the respective attribute.

We suggest that an adequate (though incomplete) state of knowledge requires the following minimum data, broadly agreed between at least two independent data sets: (1) the effects of single psychophysical variables on single color attributes (with other variables held constant), and on paired attributes, and on all three attributes simultaneously (note these correspond to Eq. 1 above); and (2) the effects of paired psychophysical variables (e.g., variable dominant wavelength at various purities) on single color attributes, and on paired attributes, and on all three attributes simultaneously. Given an adequate database on the above effects of the psychophysical variables on the color attributes, the next research requirement may be to determine the interrelationship of color attributes, from the above or from independent experiments to find how one attribute affects another whilst the third attribute is held constant (i.e., relations in Eq. 2). Some of the simpler effects are already predicted by existing color appearance models [4], but from scarce or conflicting data.

Plainly, the many effects and their experimental parameters need clear naming and classification to differentiate them and to allow rigorous repeatability of experiments [13,49].

### How Do Six Color Attributes Fit into Three Dimensions of Color?

A question arises when a color may have been defined in color appearance experiments or formulas by four or more attributes. Color has been regarded traditionally as 3-dimensional, and treated as such by various color-order systems though their color attributes may have different names. For example, the Natural Color System [2] uses whiteness/blackness rather than lightness. The Rosch and the Luther-Nyberg 3-dimensional color solids (page 184 of [2]) represent optimal color stimuli in the CIE x, y, Y color space. Fig. 1 outlines a simpler type of 3-dimensional color solid, an easily visualized concept from the Munsell color order system, whose main color attributes are lightness (or Value), hue, and chroma.

The question arises, Is the traditional 3-dimensional color solid still relevant when modern color appearance attributes number more than three? A higher number of attributes in defining a given color makes for accuracy and is fine in theory, but does a higher number than three make sense in 3-dimensional color? Is a higher number than three logically possible in 3-dimensional color? Crucial to the problem are the definitions of “dimension” and “attribute”. Traditionally, three dimensions meant three perceivable attributes, so “dimension” and “attribute” were virtually interchangeable. That no longer remains the case with (at least) six color attributes.

A reasonable solution is to allocate a group of related attributes (such as chroma, saturation, colorfulness) to each dimension. On this basis, and given a color defined by six attributes, we (co-authors) and colleagues were able to visualize a color solid as 3-dimensional, if each dimension represented only one attribute at a time. Mathematically and colorimetrically, that is an acceptable concept of 3-dimensional color if (1) each dimension is tied to a group of similar, interchangeable attributes (e.g. lightness and brightness), and (2) if each dimension represents only one attribute at a time. This is because an observer can perceive, or make estimates of, only one attribute at a time, just as we can think only one thought at a time.

In this way, color remains 3-dimensional but each dimension can represent a different attribute at different times. This type of 3-dimensional color is schematized in Fig. 1, where each dimension is tied to a group of similar interchangeable attributes.

## Classification and Nomenclature

Psychophysics is the study of relations between measurements of physical or physics-based stimuli and the evoked sensations [4]. The physical stimulus in color vision comprises radiant power and wavelength (Fig. 1). Because these do not directly and proportionally evoke color sensation, the visual stimulus is usually measured in terms of the so-called psychophysical variables, whose relationships intermediate to physics and psychology are admirably schematised in Fig. five of [1]. As shown in Fig. 1, the main psychophysical variables are: luminance (the photometric magnitude corresponding to the radiometric magnitude named radiance), dominant wavelength, and purity. The variable purity arises when two or more wavelengths simultaneously strike the eye, forming an admixture (even a white) that is less pure in hue than monochromatic radiation. Colorimetric purity and excitation purity are defined in [2,5]; the former purity, unlike the latter, is independent of the color space, but the latter purity is a better approximation of chromaticness. These three variables (luminance, dominant wavelength, and purity) are accepted in general convention [1,16] as the basic psychophysical stimulus variables for both unrelated (e.g. aperture) colors and related (e.g., object or surface) colors.

The main attributes of color appearance for all modes of color appearance are lightness, brightness, hue, and chromaticness [2,4]. The latter may be perceived as chroma, colorfulness, or saturation. These six attributes are used in color appearance models to define or predict colors. But many observers find it difficult to perceive or even conceptualize all three attributes of chromaticness and tend to favor or perceive one more than others. The six attributes together with the three psychophysical variables give 18 possible effects, as shown in Table 1. These effects are clearly the most important effects because, to understand the psychophysical variables’ influences on color appearance, it is first necessary to know the effect of each variable on each attribute.

All color order systems employ 3-dimensional color. For most purposes, a minimum of three attributes may be used to define color; e.g., brightness, hue, and saturation are commonly perceived for unrelated colors, while lightness, hue, and chroma are commonly perceived attributes for related colors [4]. As mentioned above, 3-dimensional color remains relevant to the six attributes, since the latter divide into 3 groups of similar attributes, each group tied to one of the dimensions, with each dimension representing only one attribute at a time.

It is proposed to name all effects by first their psychophysical variable and second the effect’s color attribute. For example, the effect of purity on hue (the well-known Abney effect) will be named the Purity-on-hue effect. The name is self-explanatory, unlike naming effects after the discoverer(s). The name may be abbreviated for use in tables etc. As Table 1 shows, the effects’ abbreviated names will be based wherever possible on the initial letters of the psychophysical variables (L, D, P in upper case) and color attributes (l, b, h, c, f, and s, in lower case). For example, the abbreviation of the Purity-on-hue effect is spelt Ph.

The names for effects of combined psychophysical variables on combined attributes become awkwardly long. For example, the well-known effect of variables Dominant wavelength and Purity on chromaticity (h and c) is the Dominant wavelength-Purity-on-hue-chroma effect. This is more easily referred to in its abbreviated form as DPhc. Hence the abbreviated names will be commonly used from hereon. The advantage of using the preposition “on” is that the psychophysical variables (e.g., Dominant wavelength-Purity) are clearly separated from the affected attributes (e.g., hue-chroma) by the word “on”. This is particularly the case in spoken language. However, the written abbreviations need no such separation since capital letters clearly differentiate the psychophysical variables from the affected attributes.

The effect name specifies the psychophysical variable under consideration (e.g., L) and implies the remaining psychophysical variables are held constant. Occasionally radiance (a radiometric or physical magnitude) has been used as the measured stimulus rather than luminance (a photometric magnitude) and this should be also noted in the proposed effect description. In this relatively unusual case, the effect name should be, for example, Radiance-on-hue (Rh).

Note that Ph (in the proposed naming system) immediately indicates the stimulus and effect (as P and h, respectively), whereas “Abney effect” lacks descriptive value. Further, the latter method is clumsy in that two or three discoverers may be involved, e.g., the Bezold-Brucke effect, or the discoverers may be controversial or regional; e.g., the Abney effect in USA is named the Aubert-Abney effect in Europe. Currently, some effects either lack names or are named in a variety of potentially confusing ways; for example, the Helmholtz-Kohlrausch effect [4], whereby brightness depends on luminance and chromaticity, incorporates the so-called Brightness:Luminance effect or B:L ratio [2,4]. Only a few traditional names (e.g., “Crispening” and “Spreading”) immediately express the effect, but fail to indicate the stimuli and the affected attributes.

Many effects on color occur from combinations of psychophysical variables (e.g., the effect of dominant wavelength and purity on perceived lightness is the well-known Helmholtz-Kohlrausch effect), or from combinations of attributes, e.g., the effect of luminance on hue and chroma, shown as chromaticity ellipses in CIE diagrams [50,51]. Table 2 lists the proposed classes of effects, for any given light source. Classes are formed as follows: Classes I-III represent the effects of single psychophysical variables on single color attributes (Class I), on a pair of attributes (Class II), and on a triplet of attributes (Class III); Classes IV-VI represent the effects of pairs of psychophysical variables on single color attributes (Class IV), on pairs of attributes (Class V), and on triplets of attributes (Class VI); Classes VII-IX represent the effects of triplets of psychophysical variables on single color attributes (Class VII), on pairs of attributes (Class VII), and on triplets of attributes (Class IX); as shown in Table 2. The numbers of types of effect per class are shown in parentheses. The total number of possible combinations of 3 psychophysical variables and 6 color attributes (the maximum perceivable) is 287 effects. But, as already discussed (section “Preliminary Considerations”), this number reduces to 161 viable effects when omitting a number of psychologically impossible effects comprising 4 or more attributes at one time in a 3-dimensional color. Hence Table 2 represents the 161 total viable effects. Types of effect and their (abbreviated) names for some example classes are detailed in Tables 1, 3, and 4.

**Table 2:** Classification of the effects of 3 psychophysical variables on a maximum 6 color attributes for all modes of appearance by singles, pairs, and triplets of psychological variables and of color attributes.

		Psychophysical Variables (3)		
		singles	pairs	triplets
**Color Appearance Attributes**	**singles**	Class I (18)	Class IV (18)	Class VII (6)
	**pairs**	Class II (33)	Class V (33)	Class VIII (11)
	**triplets**	Class III (18)	Class VI (18)	Class IX (6)

Parentheses show the number of effects per class for the total 6 color attributes (lightness, brightness, hue, chroma, colorfulness, saturation), giving 161 total effects, i.e., the mathematically possible 287 effects less 126 psychologically impossible effects (see section “Preliminary Considerations”). The 161 total allows for maximum 3 color dimensions, and one attribute per dimension at one time. That is, the triplet may be any 3 of the 6 attributes *providing* only one of the 2 attributes brightness and lightness are in the same pair or triplet, and *providing* only one of the 3 chromatic attributes chroma, colorfulness, and saturation are in the same pair or triplet.

**Table 3:** Class II effects of single psychophysical variables on pairs of color attributes for all modes of appearance (i.e., total six color attributes).

*Class II*	L	D	P
*lightness & hue*	Llh	Dlh	Plh
*lightness & chroma*	Llc	Dlc	Plc
*lightness & colorfulness*	Llf	Dlf	Plf
*lightness & saturation*	Lls	Dls	Dls
*brightness & hue*	Lbh	Dbh	Pbh
*brightness & chroma*	Lbc	Dbc	Pbc
*brightness & colorfulness*	Lbf	Dbf	Pbf
*brightness & saturation*	Lbs	Dbs	Pbs
*hue & chroma*	Lhc	Dhc	Phc
*hue & colorfulness*	Lhf	Dhf	Phf
*hue & saturation*	Lhs	Dhs	Phs

The mathematically possible total of 45 effects is reduced in this table to 33 psychologically viable effects by omitting (see section “Preliminary Considerations”) 12 impossible attribute combinations, e.g., an effect on both chroma and saturation at one time, or on lightness and brightness at one time.

**Table 4:** Class VI effects of paired psychophysical variables on triplets of the six color attributes.

*Class VI*	*L & D*	*L & P*	*D & P*
*lightness, hue, chroma*	LDlhc	LPlhc	DPlhc
*lightness, hue, colorfulness*	LDlhf	LPlhf	DPlhf
*lightness, hue, saturation*	LDlhs	LPlhs	DPlhs
*brightness, hue, chroma*	LDbhc	LPbhc	DPbhc
*brightness, hue, colorfulness*	LDbhf	LPbhf	DPbhf
*brightness, hue, saturation*	LDbhs	LPbhs	DPbhs

As shown, the number of psychologically viable triplets of color attributes is six, and thus the total number of viable effects is 18.

### Naming Temporal and Other Experimental Parameters

**Fig 2:**
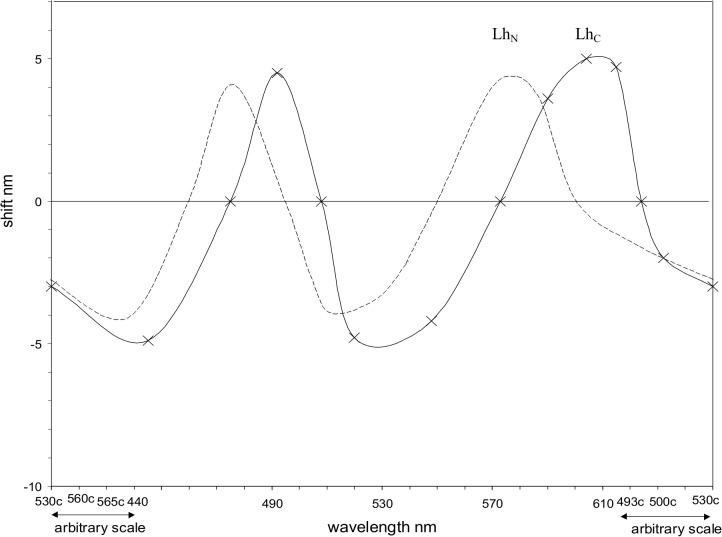
Graphs of the Luminance-on-hue (or Bezold-Brucke) effect for the no-contrast mode (labeled as Lh_N_) and for the contrast mode (Lh_C_), each for two subjects and illuminant D65; after Pridmore [20]. The ordinate shows the wavelength shift (nm) of the hue attribute. The curves are similar but Lh_N_ is shifted to shorter dominant wavelength by 10–25 nm.

In addition to the basic name of the effect (examples in Tables 1–4), effect names should also briefly indicate (in parentheses) other relevant experimental parameters such as spatial, temporal, or chromatic. For example, a parameter whose importance is sometimes underestimated is the temporal mode of observation. That is, samples may be viewed either singly (with an interstimulus interval, to remove the effect of any after-image) or simultaneously. For example, the difference between these two modes has been reported in [20], where their influences were shown to be very different in the Luminance-on-hue (Bezold-Brucke) effect as shown in Fig. 2, and therefore potentially in other effects. The difference is most plainly seen in observing a pair of hues singly and then simultaneously, when each hue can change markedly due to chromatic induction (also known as simultaneous contrast). The first mode may be termed the no-contrast mode (for stimuli observed singly, with a sufficient interstimulus interval) and the second may be termed the contrast mode (for simultaneous or, the same thing, immediately successive stimuli).

The effect’s name should specify, in parentheses, experimental parameters such as illuminant, luminance level, size, surface color, etc. For example, an effect’s name may be Dominant wavelength-Purity-on-lightness abbreviated to DPl. The complete name, including experimental parameters, may be “DPl (contrast mode, D65, 50 cd/m^2^, 2° field, object color).” This name gives the essentials of the experiment and the effect. When using the CIEDE2000 color-difference formulas under non-reference conditions [14], the parentheses may specify those conditions, which include illuminant, illuminance, background, viewing mode, sample size and separation, etc., all of which substantially affect the perception of color differences [49].

### General

The above classification system is relatively simple, considering the large number of possible physical and other variables that may impact the illumination and chromaticity of scene and surrounds from moment to moment. The proposed system (like color-order systems and color appearance models and spaces) assumes the simplest situation where the illumination, surround/background, and viewing conditions are held constant.

The benefits of classification include comparison, standardization, and analysis. An immediate lesson from Class I is that not all color appearance systems can allow for the 18 basic effects in Table 1. For example, in the DIN system [2], hue is represented as constant with varying purity; and in NCS, maximum chromaticness is constant with varying dominant wavelength. Hence DIN, as a color appearance system, is unable to represent the effect of Purity-on-hue), and NCS is unable to represent the effect of Dominant wavelength-on-chroma (or saturation or colorfulness). In fact, all color-normal people perceive that hue usually varies with purity, and that chromaticness (e.g., chroma) usually varies with dominant wavelength; e.g., maximum relative chromaticness for blue 460 nm is perceived as far higher than that for yellow 575 nm (Figures I-VII(6.6.1) of [2]).

The large number of effects emphasizes the need for systematic classification and nomenclature. A survey of known effects in the literature indicates all can be covered by the proposed classification system. The effect name, e.g., Dominant wavelength-on-hue (Dh), specifies the type (and indirectly the class) of effect, and though the effect name is simple and limited in variety, the naming system may include (simply by use of parentheses) the indication of most experimental parameters to allow for the infinite variety of effects.

The proposed system is intended for the effects of psychophysical (or physics-based) variables on color attributes but is easily extended to the effects of color attribute(s) on other attributes. In naming the effects of one color attribute on another, the same principles apply except a comma should separate the stimulus from the affected attribute; e.g., l,h denotes the effect of lightness on hue, and lc,h denotes that of lightness and chroma on hue.

### Traditional Names for Effects

Table 1 shows how some of the traditionally named effects are represented in the proposed classification. For example, Ll or Lb represents the Stevens effect and similar effects such as the Bartleson-Breneman Equations; the difference between such similar effects would be indicated by the experimental parameters given in parentheses. Dl or Db represents the Helmholtz-Kohlrausch effect (or Brightness:Luminance ratio). Another influence of the Helmholtz-Kohlrausch effect is that of varying Purity-on-lightness or brightness, i.e., Pl and Pb. Lf represents the Hunt effect (of Luminance-on-colorfulness). Lh represents the Bezold-Brucke effect. Ph represents the Abney effect.

Simultaneous contrast, e.g., from experiments on the effect of background luminance and/or dominant wavelength on the color of a sample, may be represented by Luminance-on-lightness (Ll) or Dominant wavelength-on-hue (Dh), or by Luminance-Dominant wavelength-on-lightness-hue (LDlh), depending on the experimental parameters (to be indicated in parentheses).

Crispening, which is similarly an effect of background upon samples’ perceived lightness or chromaticity, may be similarly represented as an effect of the background’s psychophysical variables on color attributes, e.g., Luminance-on-lightness (or Ll), as the general *type* of effect. But the *detailed* effect, and its difference from other Ll effects, would be indicated in the detail in parentheses: For instance, “(…background, 30 cd/m^2^; sample #1, 20 cd/m^2^; sample #2, 40 cd/m^2^; etc …).” In the case of chromatic Crispening effect, it may be represented as Dominant wavelength-Purity-on-hue-chroma (or DPhc).

However, if the experiment’s background were defined by the same color appearance terms (e.g., Munsell Value, chroma, and hue angle) as defined the samples, it would be appropriate to name the effect in color appearance terms, for example: (a) lightness-on-lightness, abbreviated as “l, l” (as described above), or (b) chromaticity-on-chromaticity (or hue-chroma-on-hue-chroma), or “hc, hc.”

The effects of single variables L, D, or P, on chroma have no common names but they are amply demonstrated in Munsell chroma data [2].

### Variable Light Source

The three psychophysical variables (L, D, P) and their effects on color attributes are dependent on a given light source and its neutral chromaticity. Of course, zero P (or achromacy) for a given light source does not necessarily represent zero P for any other source. Consequently, variable light source may possibly be considered a fourth psychophysical variable. This factor concerns chromatic adaptation that serves color constancy, an important feature of vision. However these issues are beyond adequate treatment in the present paper, which focuses on the three traditional psychophysical variables. If some other variable than CIE chromaticity coordinates is required to represent variable light source, we suggest that illuminant/source correlated color temperature (CCT) is suitable for chromaticities close to the Planckian locus. The related effects may be named those of Illuminant-on-(attribute), e.g., Illuminant-on-hue.

## Limitations of Current Data

Data on some known effects are scarce, nonspecific, or conflicting. Some well known relationships such as between luminance and brightness remain under discussion [52]. These inadequacies probably derive to some degree from lax classification and naming. If an effect lacks a formal classification and name, it may well be confused with other (un-named or similarly named) effects which in fact have quite different stimuli or parameters. The proposed system differentiates similar effects, and indicates the effect and its parameters more clearly than currently. For example, “Abney effect” does not differentiate between the original parameters (a stimulus diluted by addition of white, and thus increased luminance) or the correct parameters (both reference and test stimuli at constant luminance). In the proposed system, Ph means the effect of Purity-on-hue with other variables held strictly constant, whereas the original Abney effect may arguably be denoted as LPh since both luminance and purity were variable.

Data on the temporal modes’ difference in effects are scarce since the modes are rarely differentiated. Three examples suffice. Most data on the Lh (or Bezold-Brucke) effect refer to the contrast mode, but the contrast and no-contrast modes of Lh have been compared and differentiated at least once [20]. Fig. 2 shows the two effects give similar curves but the Lh (no-contrast) curve is shifted to shorter dominant wavelength, thus shifting curve peaks to nulls and vice-versa. A second example is the Ll (or Stevens) effect [36], where an image containing various luminances is observed altogether, demonstrating that higher luminance gives exponentially greater lightness contrast. In the no-contrast condition, however, where one of the various lightnesses (say, a white patch) is observed singly in one level of illuminance and later in a higher level, the effect of luminance on lightness is minimal (the patch still appears white). A third example is the Dh (contrast) effect. If samples of different dominant wavelength are observed singly, they appear quite different hues than if observed simultaneously in pairs.

**Fig 3:**
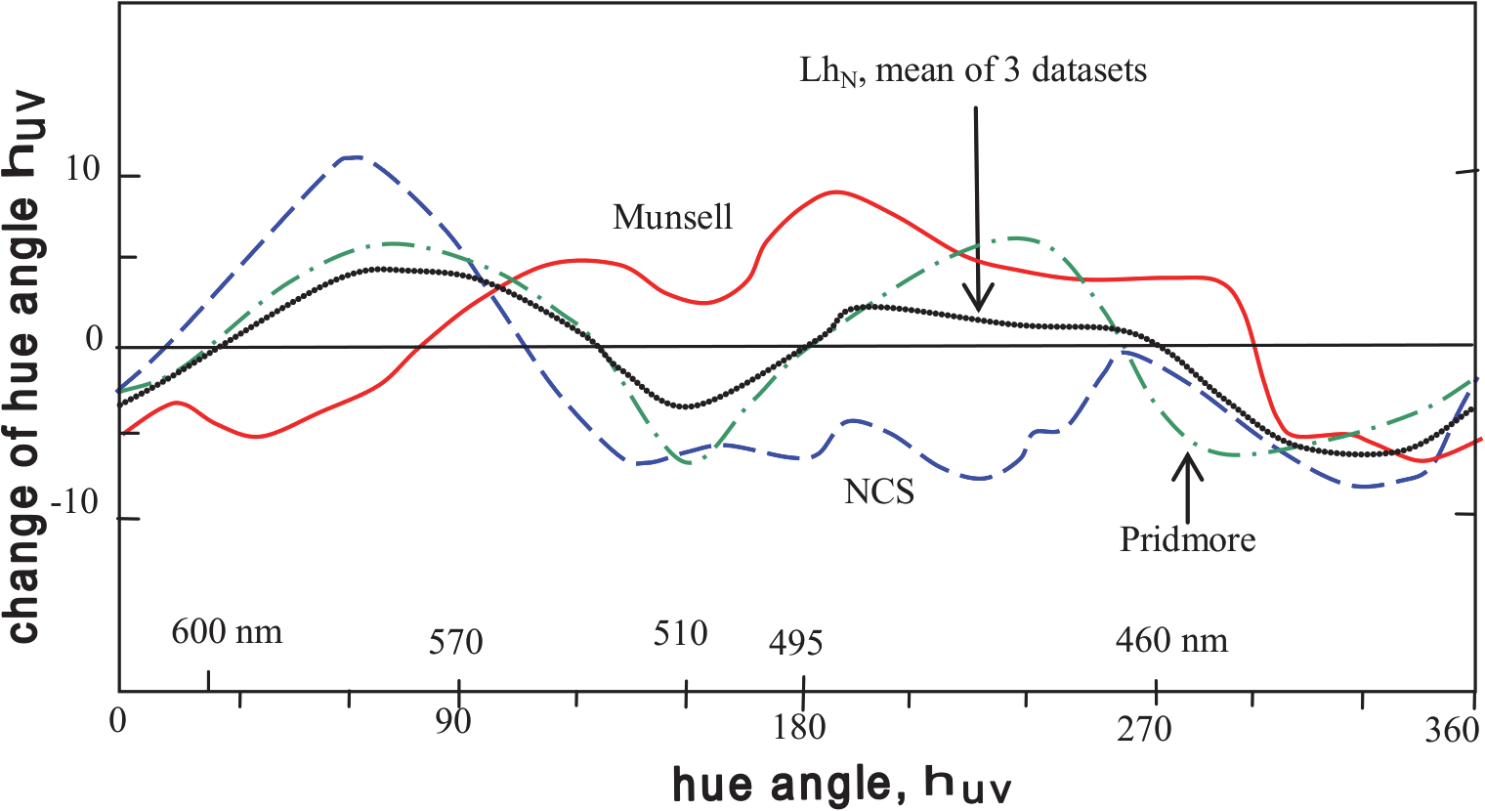
Munsell (solid line) and NCS (dashed) data for the Luminance-on-hue (no-contrast) effect; after Hunt [35]. Dash-dot line: Pridmore [20] data for Lh (no-contrast) effect as in Fig. 2. Dotted line: Mean of the three curves; the curve is labeled Lh_N_ to indicate the no-contrast mode.

Munsell and NCS data on the Luminance-on-hue effect (for no-contrast mode) exemplify conflicting data. Fig. 3 is adapted from Hunt [53] who shows the two data sets give largely opposed curves. The Pridmore curve [20] also differs from the Hunt curves. Plainly, the Lh (no-contrast) effect needs clarification. A mean of the three curves resembles the Pridmore curve, suggesting it is a suitable compromise function until Lh (no-contrast) is clarified.

**Fig 4:**
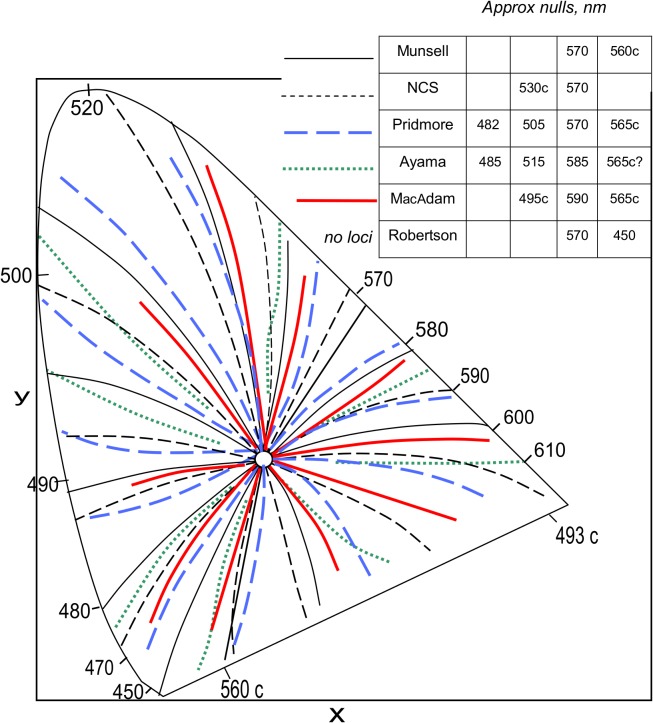
Five data sets of constant-hue loci, reflecting the Purity-on-hue (or Abney) effect in the CIE 1931 diagram for illuminant C (open circle). Some curvatures are exaggerated and some loci slightly shifted for clarity. Data sets differ in some parameters. Ph (contrast mode) effect is shown for Munsell data (samples at equal luminance), NCS data (equal blackness 0 *s*), Pridmore data [9] (equal luminance, mean of 31 subjects), MacAdam data [25] (equal brightness, mean of 2 subjects), and Ayama data [26] (equal brightness to 100 td reference white, mean of 2 subjects). The latter three data sets derive from different CIE diagrams or different light sources, so their loci are approximated by normalising them to center on illuminant C. **Inset Table**: Approximate nulls in dominant wavelength nm for the 5 data sets, plus that for Robertson [8] (mean of 3 subjects). Only nulls about 570–590 nm (yellow) and 450 nm-565 c (violet) are common to most sets.

Another example of confusing data in the literature is the Purity-on-hue (or Abney) effect. The various data sets (e.g., Munsell, NCS, and [8,9,25,26]) give widely varying results. Fig. 4 shows hue shifts and nulls differ substantially between data sets. The only area of general agreement is the null in the yellowish area. Yet all these data sets presume to represent the one “Abney effect”. The various data sets partly owe their different results to their different parameters (e.g., constant luminance, varying luminance, constant lightness, constant blackness, aperture color, object color, etc.).

There is clearly a lack of a consistent or standard naming system in the literature at present. Some effects are named after the discoverers, and some after the perceived effect (e.g., crispening). The current proposed classification and nomenclature system would improve this sadly confused situation, since the effect name would include the main experimental parameters. For example, the complete effect name may be Purity-on-hue (contrast mode, D65, 35 cd/m^2^, 2° field, surface color).

At present there are relatively few effects already known or under investigation so lack of a rigorous classification and naming system is not a big problem. But in future, as potential effects in Classes II-IX are investigated and then checked by other experimenters, the situation will grow sufficiently complex as to need a taxonomic discipline. However, experimental research progress is slow, and complicated by most observers’ difficulty in discriminating the saturation, chroma, and colorfulness attributes, and tending to favor one over the others [11,54].

## Conclusions

The present system of naming the effects of psychophysical variables on color attributes is inadequate and sometimes confusing. The number of possible effects is larger than generally thought and requires a systematic classification in order to specify the effects without confusion. A simple yet rigorous system of classification and nomenclature is proposed in this paper. The name for each type of effect is simple and self-explanatory. Each type of effect has infinite variations dependent on the experimental parameters. The latter may be indicated together with the effect name in the proposed system, which is sufficiently flexible to allow for the infinite variety of effects.

There are 161 possible types of effects for a given light source, assuming 3-dimensional color and one attribute perceptible per dimension at one time. Clearly, all 161 types of effects may not be reported for some decades. Class I effects are the most important, since to understand the stimulus variables’ effects on color it is first necessary to determine the effect of each psychophysical variable on each color attribute. Yet even today some Class I effects remain lacking experimental data in good agreement between data sets. Plainly, a lack of adequate data on the effects of the psychophysical variables on the color attributes is problematic to understanding and modeling color appearance and color difference formulas.

## Appendix 1 - Definitions

### Psychophysics

The scientific study of the relationships between the physical measurements and the sensations and perceptions that hose stimuli evoke (M. Fairchild [4])

The study of mental processes by quantitative methods specifically the reports of human subjects of the perceptions resulting from carefully measured light stimuli (R. Kuehni [55]).

### Colorimetry

Colorimetry is the measurement of color [4].

Colorimetry is the branch of color science, derived from psychophysical measurements of color matching functions, concerned with the numerical specification of color stimuli.

### Color attributes [4]

Hue: Attribute of a visual sensation according to which an area appears to be similar to one of the perceived colors: red, yellow, green and blue, or to a combination of two of them.

Brightness: Attribute of a visual sensation according to which an area appears to emit more or less light.

Lightness: The brightness of an area judged relative to the brightness of a similarly illuminated area that appears to be white or highly transmitting. Note: Only related colors exhibit lightness.

Colorfulness: Attribute of a visual sensation according to which the perceived color of an area appears to be more or less chromatic.

Chroma: Colorfulness of an area judged as a proportion of the brightness of a similarly illuminated area that appears white or highly transmitting. Note: Only related colors exhibit chroma.

Saturation: Colorfulness of an area judged in proportion to its brightness.
